# An exploratory study on the checkout rate of circulating tumor cells and the prediction of efficacy of neoadjuvant therapy and prognosis in patients with HER-2-positive early breast cancer

**DOI:** 10.3389/fonc.2022.966624

**Published:** 2022-08-04

**Authors:** Jinmei Zhou, Jiangling Wu, Xiaopeng Hao, Ping Li, Huiqiang Zhang, Xuexue Wu, Jiaxin Chen, Jiawei Liu, Jinyi Xiao, Shaohua Zhang, Zefei Jiang, Yanlian Yang, Zhiyuan Hu, Tao Wang

**Affiliations:** ^1^ Breast Cancer Department of Oncology Institute, the Fifth Medical Center of Chinese People's Liberation Army (PLA) General Hospital, Beijing, China; ^2^ Department of Clinical Laboratory, University-Town Hospital of Chongqing Medical University, Chongqing, China; ^3^ Medical Sciences Research Center, University-Town Hospital of Chongqing Medical University, Chongqing, China; ^4^ Department of General Surgery, the First Medical Center of Chinese People's Liberation Army (PLA) General Hospital, Beijing, China; ^5^ Chinese Academy of Sciences (CAS) Key Laboratory for Biomedical Effects of Nanomaterials and Nanosafety, CAS Key Laboratory of Standardization and Measurement for Nanotechnology, CAS Center for Excellence in Nanoscience, National Center for Nanoscience and Technology, Beijing, China; ^6^ Department of Oncology, the Fifth Medical Center of Chinese People's Liberation Army (PLA) General Hospital/Chinese People's Liberation Army (PLA) Medical School, Beijing, China; ^7^ The Second School of Clinical Medicine, Southern Medical University, Guangzhou, China; ^8^ School of Nanoscience and Technology, Sino-Danish College, University of Chinese Academy of Sciences, Beijing, China; ^9^ Fujian Provincial Key Laboratory of Brain Aging and Neurodegenerative Diseases, School of Basic Medical Sciences, Fujian Medical University, Fuzhou, China; ^10^ School of Chemical Engineering and Pharmacy, Wuhan Institute of Technology, Wuhan, China

**Keywords:** circulating tumor cells (CTCs), neoadjuvant therapy, liquid biopsy, prognostic, breast cancer, HER-2-positive early breast cancer

## Abstract

**Background:**

Neoadjuvant therapy is a standard treatment for patients with large, nonmetastatic breast cancer and may allow breast-conserving surgery after tumor downsizing while decreasing the risk of subsequent relapse. Dynamic changes of circulation tumor cells (CTCs) have a role in predicting treatment efficacy of breast cancer. However, the relationship between CTC enumeration before neoadjuvant therapy and pathologic complete response rate is still uncertain.

**Methods:**

The study was exploratory. A total of 50 breast cancer patients were enrolled in a phase II clinical study of neoadjuvant therapy for HER-2-positive early breast cancer. They were enrolled for blood draws before and after neoadjuvant therapy. We used two methods (*CellSearch* and *TUMORFISH*) to detect CTCs. We compared the sensitivity of the two systems and investigated the correlation of the enumeration on baseline CTCs with the diagnosis, prognosis, and efficacy of neoadjuvant therapy of the patients with HER-2-positive early breast cancer. We also explored the dynamic change of CTCs after neoadjuvant therapy.

**Results:**

The sensitivity of *TUMORFISHER* (27/50) method was significantly higher than that of the *CellSearch* system (15/50, *p*=0.008). The CTC numbers detected by the two detection systems were not significantly correlated with lymph node status, clinical stage, ki-67 level and hormone receptor status. Patients with ≥1 CTC before neoadjuvant therapy measured by the *TUMORFISHER* system had a significant high pCR rate (74.1% vs. 39.1%, *p* = 0.013); whereas, there was no predictive effect on pCR by CellSearch system (73.3% vs. 51.4%, *p* = 0.15). Patients with a decrease in CTCs enumeration after neoadjuvant therapy were more likely to achieve pCR than those with no change or increase in CTCs enumeration (87.5% vs 50.0%, *p* = 0.015) by the *TUMORFISHER* method. Unfortunately, there was no predictive value of CTCs enumeration for EFS before and after neoadjuvant therapy by two methods.

**Conclusions:**

Our study demonstrates that the new CTCs detection method *TUMORFISHER* system has a higher checkout rate in early breast cancer than the *CellSearch* system, and shows the opportunity of CTC enumeration as a novel assistant biomarker to predict the response of neoadjuvant therapy in patients with HER-2-positive early breast cancer.

## Introduction

Breast cancer is the most common carcinoma in global females, and distant metastasis is the leading cause of cancer-related deaths (1). Circulating tumor cells (CTCs) are "tumor cells shedding from the primary tumor or metastases into the bloodstream, either spontaneously or due to diagnostic and therapeutic procedures", which were firstly described by Ashworth in 1869 (2). Most CTCs entering the circulation would die in a short time, and only a few CTCs with high vitality and metastatic potentiality survive and form metastases under certain conditions. Therefore, detection of tumor cells in bloodstream may indicate the development or metastasis of tumor (3).

The *CellSearch* system is currently the only CTC detection system approved by the U.S. Food and Drug Administration (FDA) and China Food and Drug Administration (CFDA). Epithelial cell adhesion molecule (EpCAM) antibody-labeled magnetic beads were used to enrich tumor cells in *CellSearch* system, which defined the cells with cytomorphological tumor characteristics and phenylindole DAPI(+), cytokeratin CK(+), and leukocyte antigen CD45(-) as CTC. Although current clinical practice guidelines do not yet recommend making treatment decisions based on CTCs enumeration or phenotyping for patient with metastatic breast cancer (MBC), multiple studies have confirmed that CTCs are widespread in MBC and high baseline CTC counts based on *CellSearch* is a well-established independent prognostic factor for worsening progression-free survival (PFS) and overall survival (OS). Moreover, dynamic changes of CTCs have a role in predicting treatment efficacy (4–8). The checkout rate of CTC in patients with early breast cancer (EBC) is significantly lower than that of MBC, ranging from 21.5% to 24% as reported in previous study, and≥ 1 CTC/7.5ml blood is a poor prognostic factor for disease free survival (DFS) and OS (9–12). However, the relationship between CTC counts before neoadjuvant therapy and pathologic complete response (pCR) rate is still uncertain (13).The pCR status of patients with HER-2-positive EBC after neoadjuvant therapy has a definite prognostic value and can guide preoperative and postoperative treatment to further improve patients’ survival, so the guidelines currently recommend neoadjuvant therapy as the first choice for patients with breast tumors ≥ 2 cm or axillary lymph node metastasis (14–16). Only a few small retrospective studies have explored the predictive value of CTC counts for pCR in patients with HER-2-positive EBC so far, and the results are inconclusive (11, 17–19).

The major factor limiting the use of the *CellSearch* system for CTC detection in research and clinical practice is its low checkout rate. Therefore, many studies have focused on developing more sensitive CTC detection platforms. *TUMORFISHER* nanotechnology developed by the National Center for nanoscience and technology of the Chinese Academy of Sciences, utilizes peptide nanomagnetic particles to capture CTCs from peripheral blood, requiring only 2 ml blood per assay. Preliminary studies have shown that it can achieve a similar prognostic value to the *CellSearch* isolation system for advanced breast cancer, but there is no study to explore its sensitivity and predictive and prognostic value in EBC patients (20, 21). Based on a randomized, open-label, phase II clinical study conducted by our center to evaluate the efficacy and safety of trastuzumab combined with anthracycline or non-anthracycline-platinum as neoadjuvant therapy for HER-2-positive breast cancer (NCT 02510781), this study prospectively and dynamically collected blood samples from the enrolled patients. The objective of this study was to compare the sensitivity of the two CTC detection systems in patients with HER-2-positive EBC and explore the relationship between CTC status and the clinicopathological characteristics of the primary tumor and its predictive and prognostic values for the efficacy of neoadjuvant therapy.

## Materials and methods

### Patients

In this study, a total of 50 patients from the Fifth Medical Center of PLA General Hospital were enrolled in a phase II clinical study of neoadjuvant therapy for HER-2-positive EBC (NCT 02510781) from July 2015 to March 2018. The peripheral blood samples and clinicopathological data of these patients were collected.

Inclusion criteria: (1) Pathologically diagnosed with invasive breast cancer by core needle biopsy, and the tumor diameters≥2cmindicated by MRI or with positive axillary lymph nodes;(2) HER-2 positivity (Immunohistochemistry 3+ or FISH/CISH +); (3) The clinical data are complete, such as treatment records, imaging, surgery, and postoperative pathology. This study was approved by the Ethics Committee of the Fifth Medical Center of the PLA General Hospital, and all participating patients signed the informed consents.

Exclusion criteria: (1) Previous systemic or local therapy for breast cancer including chemotherapy; (2) metastatic disease (stage IV), bilateral breast cancer, or bilateral breast cancer; (3) Other malignancies, inadequate bone marrow or renal function, impaired liver function, impaired cardiac function, uncontrolled hypertension, pregnancy, and refusal to use contraception.

The eligible breast cancer patients for baseline blood draws were newly diagnosed HER-2-positive early breast cancer and were about to accept neoadjuvant therapy. The enrolled patients provided second blood draws after neoadjuvant therapy, and the periods from baseline to follow-up ranged from 18 to 24 weeks. The responses at the follow-up visit and the best overall responses were both assessed by Response Evaluation Criteria in Solid Tumors (RECIST) 1.1 guidelines.

### CTC detection methods

Blood (7.5 mL) was collected into CellSave Preservative Tubes, stored at room temperature, and processed within 24h using CellSearch system for CTC enrichment and enumeration. The *CellSearch* system (Johnson & Johnson, Veridex, USA) is a semi-automated technique that enriches EpCAM-expressing cells in blood by immunomagnetic beads labeled with anti-EpCAM antibody. The enriched samples are subsequently fluorescently labeled with the indicated antibodies for CD45 and a panel of cytokeratin antibodies, and the cells with CK^+^/DAPI^+^/CD45^-^ are adjudicated as CTCs.

Blood (2 mL) was collected in specialized CTCs preservation EDTA tube, stored at room temperature, and processed within 24h using *TUMORFISHER* detection platform for CTC enrichment and enumeration. The *TUMORFISHER* technology developed by the National Center for Nanoscience of the Chinese Academy of Sciences mixes peptide-modified magnetic nanoparticles that specifically recognize EpCAM on the surface of CTCs with blood samples, captures CTCs in blood by magnetic force, and fixes cells with 4% paraformaldehyde at room temperature. The captured cells are then identified by the immunofluorescence staining method, in which DAPI^+^/CK^+^/CD45^-^ and cells conforming to the morphology of tumor cells are determined as CTCs. The Cut-off values are shown below:

*CellSearch* system: ≥1 CTC/7.5ml peripheral blood was determined as CTC positive.

*TUMORFISHER* method: ≥1 CTC/2ml peripheral blood was determined as CTC positive.

The blood samples of 50 patients were simultaneously detected by the *CellSearch* and the *TUMORFISHER* system.

### Neoadjuvant therapy

Patients received epirubicin (75 mg/m^2^) + docetaxel (75 mg/m^2^) + trastuzumab (initially 8 mg/kg followed by 6 mg/kg) for 3~4cycles, followed by docetaxel (75 mg/m^2^) + trastuzumab (6 mg/kg) for 3~4cycles (ATH-TH group), or docetaxel (75 mg/m^2^) + carboplatin (AUC=6) + trastuzumab (initially 8 mg/kg followed by 6 mg/kg) for 6 cycles, 21 days as one cycle. After neoadjuvant therapy, patients received surgery, continued trastuzumab for 1 year and received adjuvant radiotherapy and endocrine therapy according to treatment guidelines. Up to now, there’s no standard treatment for the patients who achieve PD (Progressive disease) during neoadjuvant therapy. We recommend that patients who achieve PR (partial response) or SD (stable disease) complete neoadjuvant therapy as planned adjust systemic therapy (e.g.: Anti-HER2 targeted therapy second-line drug, T-DM1 or TKIs) or switch to surgical therapy for patients with disease progression according to clinical practice promptly and cautiously.

### Efficacy evaluation

The clinical efficacy of neoadjuvant therapy was evaluated according to RECIST 1.1 criteria. Surgical specimens were assessed for pathological complete response which was defined as no invasive residual cancer in primary breast and axillary lymph nodes, allowing residual intraductal carcinoma (ypT0/ IS ypN0; pCR). Event-free survival (EFS) was defined as the time from randomization until the date of the first occurrence of one of the following events: disease progression, metastasis, or death from any cause. During neoadjuvant therapy, clinical breast examinations were performed prior to each cycle dosing, and patients underwent mammography, ultrasonography, and magnetic resonance imaging (if clinical practice required) every two cycles until surgery. Clinical assessments for disease recurrence occurred every 3 months from the date of surgery to year 2, then every 6 months to year 5, and annually thereafter to year 10, including physical examination, blood, ultrasound, chest X-ray, bone scan as needed.

### Statistical analysis

SPSS 20.0 was used for the statistical analysis of the data. Qualitative data were expressed as sample rate or frequency, and differences between groups were compared by χ^2^ or Fisher's exact test. Kaplan-Meier curves were used to analyze patients' EFS. All statistical tests were two-sided tests, and a *p*-value less than 0.05 was considered statistically significant.

## Results

### Patients

As shown in **Table 1**, the median age of 50 patients was 49 years (range, 28~66 years). According to the 7^th^ of the AJCC breast cancer staging criteria, the number of patients with stages I, II, and III was 2 (4.0%), 33 (66.0%) and 15 (30.0%) respectively. 23 (46.0%) patients were ER positive; 14 (28.0%) patients were PR positive, and 42 (84.0%) patients wereKi-67≥30%. Up to September 2021, median follow-up was 59.2 months (range, 23.5~72.8 months). 29 (58.0%) patients achieved pCR after surgery; 1 (2.0%) patient developed disease progression during neoadjuvant therapy; 3 (6.0%) patients developed metastasis after surgery; and 1 (2.0%) patient died from other causes without metastasis.

**Table 1:** Baseline characteristics of patients (n = 50).

Feature	n (%)
Age (years)	
Median (range)	49 (28-66)
Clinical stage	
I	2 (4.0%)
II	33 (66.0%)
III	15 (30.0%)
Axillary lymph node status	
Negative	14 (28.0%)
Positive	36 (72.0%)
ER/PR status	
Negative	27 (54.0%)
Positive	23 (46.0%)
Ki-67 index	
<30%	8 (16.0%)
≥30%	42 (84.0%)
Neoadjuvant scheme	
ATH	27 (54.0%)
TCbH	23 (46.0%)
pCR rate	
ATH group	17 (63.0%)
TCbH group	12 (52.2%)

### CTCs checkout rate of two detection methods

**Figure 1:**
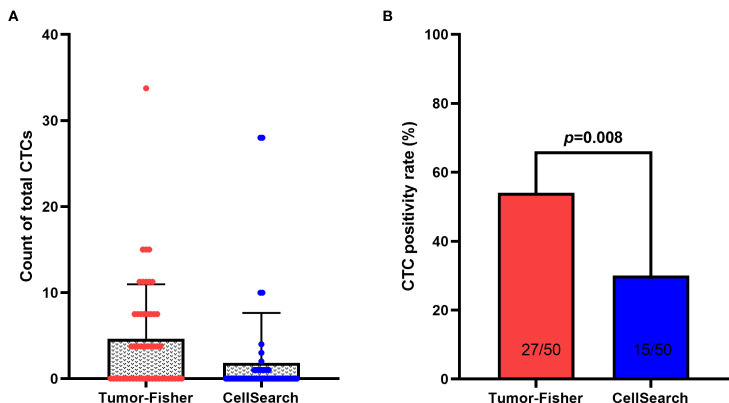
**(A)** CTCs detection by TUMORFISHER and *CellSearch* methods in 50 patients. **(B)** The checkout rate of CTCs detection by TUMORFISHER and *CellSearch* in 50 patients.

Twenty seven (54.0%) patients had ≥1 CTC/2ml detected by the *TUMORFISHER* system, and the median number of CTCs was 2/2ml (range, 1-9/2ml). 15(30.0%) patients had ≥1 CTC/7.5ml detected by the *CellSearch* system, and the median number of CTCs was 1/7.5ml (range, 1-28/7.5ml). The sensitivity of CTC detection by the *TUMORFISHER* method was significantly higher than that of the *CellSearch* system (*p*=0.008) (**Figure 1**) (**Table 2**). The CTC levels detected by the two detection systems were not significantly correlated with lymph node status, clinical stage, ki-67 level and hormone receptor status. (**Table 3**).

**Table 2:** Checkout rate of CTCs by TUMORFISHER and CellSearch in 50 patients.

CellSearch system	TUMORFISHER system		Total	
	Positive	Negative		
Positive	12	3	15 (30.0%)	
Negative	15	20	35 (70.0%)	
Total	27 (54.0%)	23 (46.0%)	50 (100%)	P=0.008

**Table 3:** Relationship between CTC levels and clinicopathological characteristics in 50 patients.

CTC status	Lymph node status		Clinical stage		Ki-67 levels		Hormone receptor	
	No	Yes	Ⅰ/Ⅱ	III	≤30%	>30%	Negative	Positive
*CellSearch* system									
Positive	2 (14.3%)	13 (36.1%)	9 (25.7%)	6 (40.0%)	2 (25.0%)	13 (31.0%)	8 (29.6%)	7 (30.4%)
Negative	12 (85.7%)	23 (63.9%)	26 (74.3%)	9 (60.0%)	6 (75.0%)	29 (69.0%)	19 (70.4%)	16 (69.6%)
χ^2^ value	1.365		0.454		0		0.004	
*P* value	0.243		0.501		1		0.951	
TUMORFISHER system									
Positive	6 (42.9%)	21 (58.3%)	18 (51.4%)	9 (60.0%)	6 (75.0%)	21 (50.0%)	14 (51.9%)	13 (56.5%)
Negative	8 (57.1%)	15 (41.7%)	17 (48.6%)	6 (40.0%)	2 (25.0%)	21 (50.0%)	13 (48.1%)	10 (43.5%)
χ^2^value	0.972		0.311		0.834		0.109	
*p* value	0.324		0.577		0.361		0.741	

### Predictive value of CTC levels before neoadjuvant therapy for pCR

Patients with ≥1 CTC before neoadjuvant therapy measured by the *TUMORFISHER* system had a significant high pCR rate (74.1% vs. 39.1%, P = 0.013); CTC levels detected by the *CellSearch* system did not show a predictive value for pCR (73.3% vs. 51.4%, P = 0.15) (**Table 4**).

**Table 4:** Relationship between CTC levels before neoadjuvant therapy and pCR in 50 patients.

CTC status	Pathology results, n (%)	
	pCR	non-pCR
*TUMORFISHER* system		
Positive	20 (74.1%)	7 (25.9%)
Negative	9 (39.1%)	14 (60.9%)
χ^2^ value	6.226	
*p* value	0.013	
*CellSearch* system		
Positive	11 (73.3%)	4 (26.7%)
Negative	18 (51.4%)	14 (48.6%)
χ^2^ value	2.068	
*p* value	0.15	

### Predictive value of changes in CTC numbers before and after neoadjuvant therapy for pCR

We also assessed the predictive value of dynamic changes of CTCs for pCR. After neoadjuvant therapy, CTCs detections by the *TUMORFISHER* system were performed in 40 patients. As shown in **Table 5**, patients with decreased CTCs count were more likely to achieve pCR than those with no change or increase in CTCs count (87.5% vs 50.0%, *p* = 0.015). 12 patients with ≥1 CTC detected by the *CellSearch* system at baseline underwent CTCs detection again after neoadjuvant therapy. Results showed a decrease in CTC counts in 7 patients and no change or increase in 5 patients, whereas changes in CTC levels exhibited no predictive effect on pCR (85.7% vs. 80.0%, p =1.0) (**Table 5**).

**Table 5:** The relationship between CTC dynamic changes and pCR.

CTC change	Pathology results, n (%)	
	pCR	non-pCR
*TUMORFISHER* system (n=40)		
Decreased group	14 (87.5%)	2 (12.5%)
No change/increased group	12 (50.0%)	12 (50.0%)
χ^2^ value	5.934	
*p* value	0.015	
*CellSearch* system (n=12)		
Decreased group	6 (85.7%)	1 (14.3%)
No change/increased group	4 (80.0%)	1 (20.0%)
*p* value*	1	

* is the statistical value of Fisher test.

### Predictive value of CTC status before neoadjuvant therapy for EFS:

**Figure 2:**
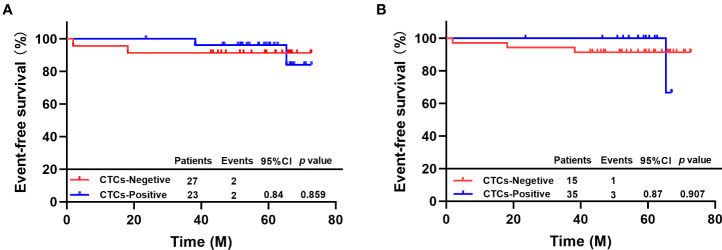
**(A)** EFS of patients with different CTC status by TUMORFISHER method; **(B)** EFS of patients with different CTC statuses by *CellSearch.*.

Two out of 27 patients with ≥1 CTC detected by *TUMORFISHER* system before neoadjuvant therapy experienced disease progression or metastasis or death. 2 out of 23 patients with no CTC at baseline experienced EFS events. The 5-year EFS rate was 96.2% in CTCs-positive patients and 91.3% in CTC-negative patients, nevertheless, there was no difference between the two groups (HR=0.84, 95% CI 0.12-5.98, *p* = 0.859, **Figure 2A**).

One out of 15 patients with ≥1 CTC detected by *CellSearch* system before neoadjuvant therapy experienced disease progression or metastasis or death. Three out of 35 patients with no CTC at baseline experienced EFS events. The 5-year EFS rate was 100% in CTC-positive and 91.4% in CTC-negative patients, there was no difference between the two groups (HR=0.87, 95% CI 0.10-7.85, p=0.907, **Figure 2B**).

**Figure 3:**
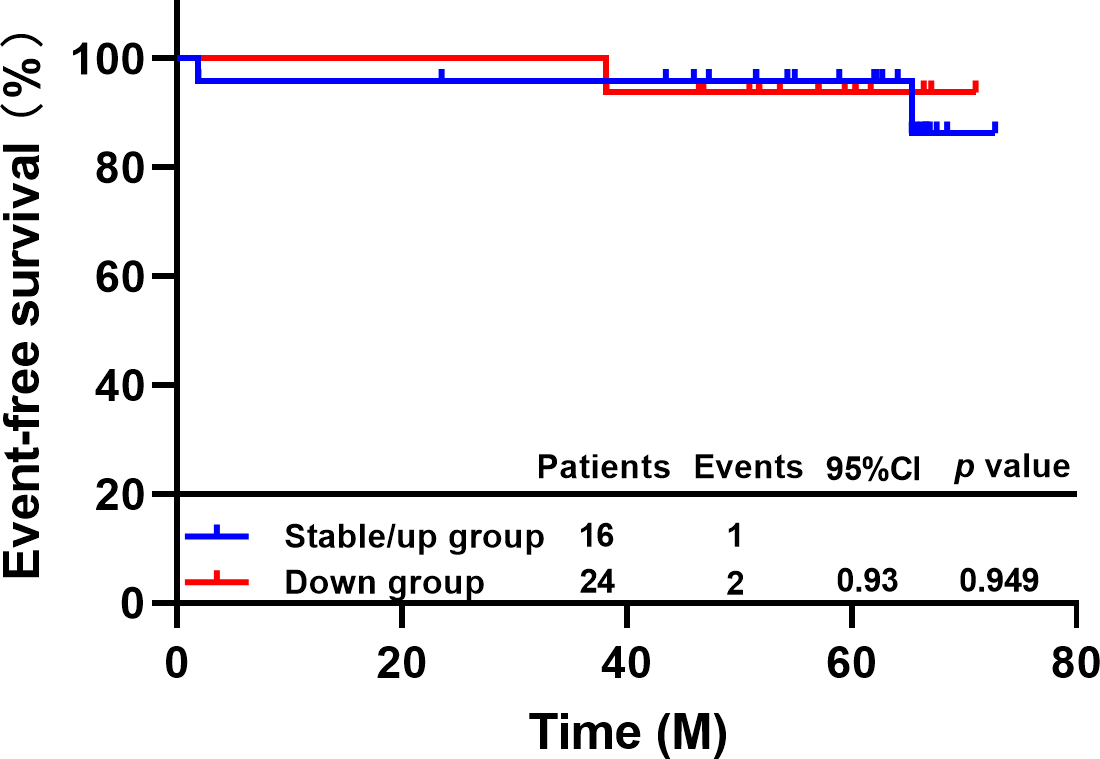
EFS of patients with different changes in CTC by TUMORFISHER method.

After neoadjuvant therapy, one of 16 patients with decreased CTC levels experienced disease progression or metastasis or death, and two of 24 patients with no change or increased CTC levels experienced EFS events. The 5-year EFS rate of patients with decreased CTC levels was 93.8%, and that of patients with no change or increase in CTC levels was 95.8%. There was no difference between the two groups (HR=0.93, 95% CI 0.09-9.91, P=0.949, **Figure 3**). In addition, there are 19.0% (n=4/21) patients occurred EFS events in non-pCR group and none of the patients (n=0/29) in pCR group. Our study showed prominent difference in the EFS between the pCR and the non-pCR groups in 6 years of follow-up results (*p*=0.016).

## Discussion

Breast cancer is a systemic disease in which tumor cells can spread early (22). Compared with traditional histological biopsy and image examination, liquid biopsy has the advantages of simple operation, non-invasiveness, and strong repeatability, which provides promise for early diagnosis and treatment, precise detection, real-time dynamic monitoring, and individualized treatment of cancer. Compared with other forms of liquid biopsies, such as circulating tumor DNA/RNA/exosomes, CTCs are intact cells and then have unique advantages in assessing genomics, transcriptomics, and proteomics. The number of CTCs in peripheral blood is rare, averaging only 1 CTC per 100,000-1,000,000 leukocytes (23). The main barriers of application of CTCs in clinical practice are unsatisfactory sensitivity of current detection technique and subsequent molecular biological analysis (24). The application of CTCs in MBC has shown independent prognostic value, but the clinical value of CTCs in EBC is rarely studied. One of the reasons is that the detection efficiency of the *CellSearch* system which is currently the only CTC detection system approved by the FDA and CFDA in EBC is unsatisfactory. Therefore, it remains necessary to explore the clinical significance of CTCs in EBC based on a more sensitive and specific detection technology.

The *CellSearch* system which used EpCAM antibody labeled magnetic beads to enrich CTCs, has disadvantages such as low sensitivity, inability to capture live cells and conduct subsequent genetic testing and medication guidance, requirement of large blood samples, and high detection cost. The *TUMORFISHER* technology developed by the National Center for Nanoscience of the Chinese Academy of Sciences uses magnetic nanobeads labeled with polypeptides which specifically recognize EpCAM to efficiently isolate CTCs in peripheral blood, and the captured CTCs are active enough for subsequent molecular biological analysis (20, 25).Our findings showed that the prevalence of CTCs detected by the *CellSearch* system was 30.0% (15/50) before neoadjuvant therapy in patients with HER-2 EBC, which is similar to the results reported in previous studies (9–12). On the other hand, the baseline CTC checkout rate of the *TUMORFISHER* system was 54.0% (27/50), which was significantly higher than that of the *CellSearch* system (p=0.008). Previous studies have shown that the prevalence of CTCs in EBC is correlated with lymph node involvement, meanwhile its relationship with histological grade, tumor size, ki-67 levels, and hormone receptor status is uncertain (11, 26). Our findings revealed that the levels of CTCs detected by the two detection systems had no significant correlation with tumor stage, lymph node status, ki-67 levels, and hormone receptor status. Our study found that the *TUMORFISHER* system was more sensitive in detecting CTCs in EBC which facilitates sequential study of CTCs, including the detection of CTC surface receptors such as ER, PR, HER-2, epidermal growth factor receptors, and programmed death-ligand 1 and the analysis of biological information such as mRNA and DNA; thereby ultimately achieving better guidance of treatment (27). Previous studies have shown that gene expression of CTCs is inconsistent with the primary tumor and has prognostic value and the analysis of ER resistance pathway signaling in CTCs can predict endocrine therapy resistance in advance (28, 29).

Multiple studies have confirmed the prognostic value of CTCs in patients with MBC and the predictive value of its dynamic change on treatment efficacy (4–8). There are also some studies and meta-analyses showing that CTC is a poor prognostic factor for DFS and OS in EBC patients (9, 10, 13).But the prognostic value of CTC after neoadjuvant therapy, the relationship between CTC counts before and after neoadjuvant therapy and pCR, and the prognostic value of CTC dynamic change during neoadjuvant therapy are still inconclusive, and studies focusing on HER-2-positive subtype are especially lacking (13).GeparQuattro trial enrolled a subset of HER-2-positive patients who received anti-HER-2 targeted therapy and found that the checkout rate of CTCs was 21.6% before neoadjuvant therapy and decreased to 10.6% after neoadjuvant therapy. Furthermore, those who were CTC positive before neoadjuvant therapy had a poor prognosis, the status of CTC after neoadjuvant therapy showed no prognostic value, and the CTC status at both time points had no correlation with pCR (17, 18). Subgroup analysis of the NeoALTTO trial showed that during neoadjuvant therapy, 3 of 11 CTC positive patients achieved pCR (27.3%) while 17 of 40 CTC negative patients achieved pCR (42.5%), with no significant difference between the two (P = 0.36) (19).

Our results showed that the baseline status of CTCs detected by the *CellSearch* system had no predictive value for pCR in HER-2 positive EBC, which was consistent with the results of previous studies (17, 19). Another CTC detection system, the *TUMORFISHER*, demonstrated that CTC-positive patients had a higher pCR rate (74.1% vs. 39.1%, p=0.013). the GeparQuattro, NeoALTTO and some other trials observed that patients with detected CTCs at any time point or at surgery had numerically lower pCR rate compared to those with no CTCs. So, CTC is a poor prognostic factor for DFS and OS in EBC patients. But in our study, we get the opposite result. Possible reasons are as follows: (i) A higher incidence of pCR was shown in ER-/HER2+ patients. There is a higher percentage of ER-/HER2+ patients (56.5%) in our clinic trial coincidentally, resulting in a higher pCR in CTC positive group. (ii) pCR is usually defined as the absence of invasive and non-invasive carcinoma in breast tissue. If we assume that aggressive cancer cells or CTCs may exist in these malignant tumors, so we could hypothesize that pCR could be explained by the eradication of highly proliferating tumor cells or CTCs. So, the completely neoadjuvant therapy has more significant effect on this high tumor burden CTC positive group. The disappearance of CTCs might be a new goal of treatment instead of pCR. (iii) Finally, this estimate is based on a small sample. There might be a statistical bias here, so more patients would need to be recruited to validate the predictive values that we demonstrated in this trial.

The checkout rate of CTC after neoadjuvant therapy was 25.0%, which was lower than that before neoadjuvant therapy. Patients with reduced CTC counts after neoadjuvant therapy were more likely to achieve pCR (87.5% vs. 50.0%, *p*=0.015), suggesting that dynamic monitoring of CTC with *TUMORFISHER* system during neoadjuvant therapy has the potential to predict efficacy, which was consistent with the conclusion of previous CTC studies in metastatic breast cancer (6, 7).Our findings revealed that neither the baseline nor the dynamic changes of CTC detected by the *CellSearch* system and *TUMORFISHER* had prognostic value, which may be related to the favorable prognosis and low recurrence risk in patients with HER-2-positive early breast cancer who received standard treatment including anti-HER-2 therapy in this study. After a median follow-up of 59.2 months, only four patients had EFS events. And the results suggest that these patients acquired pCR after complete neoadjuvant therapy is not prone to occur EFS events (e.g.: disease progression, metastasis, or death from any cause).

To the end, a few limitations should also be concerned in the present study. First of all, the aforesaid clinical significance of CTC detection in HER-2 positive EBC are based on small sample size, so more patients would need to be recruited to validate the predictive values that we demonstrated in this trial. Furthermore, the biological information carried by CTC counts is limited, so predictive significance of CTC-based HER2 phenotyping during neoadjuvant therapy should be investigated in our subsequent work. In the future, long term follow-up with regular blood draws should also be considered before these patients are assessed as metastatic disease. If so, more rigorous thoughts about the dynamic changes of CTC-HER2 phenotype may be revealed, which is important for the decision making through personalized longitudinal evaluations.

## Conclusions

The present study shows the opportunity of CTC enumeration as a novel assistant biomarker for predicting neoadjuvant therapy response in patients with HER-2-positive early breast cancer. Study shows that the new CTC detection method, *TUMORFISHER* system, has a higher CTC checkout rate in EBC than the *CellSearch* system and then provides a good foundation for subsequent molecular bioinformatics analysis. Baseline and dynamic changes in CTCs count during neoadjuvant therapy for HER-2-positive EBC preliminarily show predictive value for efficacy. Nonetheless, our findings need to be validated in a larger sample size, ideally in prospective trials.

## Data availability statement

The original contributions presented in the study are included in the article/Supplementary Material. Further inquiries can be directed to the corresponding authors.

## Ethics statement

The studies involving human participants were reviewed and approved by the Ethics Committee of the Fifth Medical Center of the PLA General Hospital (NCT 02510781). The patients/participants provided their written informed consent to participate in this study.

## Author contributions

JZ, JW, XH, PL, HZ and XW conceptualized, carried out and interpreted the main experiments. JZ, JW, XH, JC, JL, and JX was the main contributors to the investigation, date curation, formal analysis of the work. TW, XH and ZJ coordinated the enrollment of participants and provided the corresponding clinical. YY, ZH and TW supervised, administrated, and supported the project. JZ and JW wrote the original draft. All authors reviewed and edited the original draft, and approved the final manuscript.

## Funding

This study was supported by National Natural Science Foundation of China (Grant No. 81572597, 32027801, 31870992,21775031), Beijing Municipal Natural Science Foundation (Grant No. 7192198), the Strategic Priority Research Program of Chinese Academy of Sciences (Grant No. XDB36000000, XDB38010400), the Foundation of Chongqing Municipal Education Commission (Grant number: HZ2021006), CAS-JSPS(Grant No. GJHZ2094), Research Foundation for Advanced Talents of Fujian Medical University (XRCZX2017020, XRCZX2019005), the Scientific Research Funding (account number 2019JC05) and Talents Project of University-Town Hospital of Chongqing Medical University.

## Conflict of interest

The authors declare that the research was conducted in the absence of any commercial or financial relationships that could be construed as a potential conflict of interest.

## Publisher’s note

All claims expressed in this article are solely those of the authors and do not necessarily represent those of their affiliated organizations, or those of the publisher, the editors and the reviewers. Any product that may be evaluated in this article, or claim that may be made by its manufacturer, is not guaranteed or endorsed by the publisher.
